# Regulation of health professions education and the growth of schools in Somalia

**DOI:** 10.1186/s12909-024-06179-3

**Published:** 2024-10-19

**Authors:** Mulki Mukhtar Hassan, Amal Naleye Ali, Ifrah Ali, Zeinab Omar Mohamed, Hamza Mohamed Abdullahi, Mohamed Mustaf Ahmed, Abdirahman Khalif Mohamud, Yusuff Adebayo Adebisi, Olalekan John Okesanya, Don Eliseo Lucero-Prisno III

**Affiliations:** 1 Faculty of Medicine and Health Sciences, SIMAD University, Mogadishu, Somalia; 2 Faculty of Medicine, Somali National University, Mogadishu, Somalia; 3 Federal Ministry of Heath Somalia, Mogadishu, Somalia; 4 College of Social Sciences, University of Glasgow, Glasgow, UK; 5 Department of Public Health and Maritime Transport, University of Thessaly, Volos, Greece; 6 Department of Global Health and Development, London School of Hygiene and Tropical Medicine, London, UK

**Keywords:** Health professions education, Medical regulation, Somalia

## Abstract

**Background:**

Health professions education, encompassing training programs for medicine and surgery, nursing, midwifery, medical laboratory sciences,, and public health, along with their regulations, are pivotal to achieving universal health coverage and Sustainable Development Goals, contributing significantly to health outcomes and public trust in the healthcare workforce. However, low- and middle-income countries, especially in sub-Saharan Africa, face challenges, such as inadequate resources, outdated curricula, and weak governance. Somalia in particular grapples with a fragmented health system and a critical shortage of skilled health professionals, exacerbated by decades of civil war and political instability.

**Methods:**

This study employed a mixed-method approach that incorporated both qualitative and quantitative data collection and analysis. A comprehensive literature review was conducted along with semi-structured interviews with 44 key informants, including representatives from professional health schools and officials from the Ministry of Health. Additionally, five focus group discussions were held with young professionals and an online survey was administered to students enrolled in professional health courses. The data analysis employed descriptive for quantitative data, and thematic analysis for qualitative data, guided by the human resources for health (HRH) maturity model framework.

**Results:**

This study identified 112 health professions schools across Somalia, with a significant concentration in urban areas, particularly in Benadir. The health workforce analysis revealed a pronounced urban-rural disparity and a density of health professionals below the WHO’s recommended threshold. The focus group discussions and surveys highlighted the employment challenges faced by young physicians and students’ perceptions of their training and future employment opportunities.

**Conclusion:**

The proliferation of health professions schools without adequate quality control, the critical shortage and maldistribution of skilled health professionals, and the absence of a comprehensive regulatory framework are significant challenges facing Somalia’s healthcare system. The establishment of the National Health Professionals’ Council (NHPC) Act in 2020 marks a step towards addressing these issues. This study emphasizes the need for accreditation of health professions schools, capacity building of HRH teams, and collaboration among stakeholders to improve healthcare workforce development and regulation. Addressing urban-rural disparities and combating professional misconduct are also crucial for achieving universal health coverage and improving health outcomes in Somalia.

**Supplementary Information:**

The online version contains supplementary material available at 10.1186/s12909-024-06179-3.

## Introduction

Health professions education, encompassing training programs for medicine and surgery, nursing, midwifery, medical laboratory sciences and public health, along with their regulations, play a crucial role in achieving universal health coverage (UHC) and Sustainable Development Goals (SDGs). They contribute to improving health outcomes and well-being of the population by ensuring the availability and accessibility of qualified and competent health workers as well as promoting public trust and confidence in the health workforce [1]. However, in many low- and middle-income countries (LMICs), particularly sub-Saharan Africa, health professions education and regulations face significant challenges. These challenges include inadequate resources, outdated curricula, limited infrastructure, poor quality assurance, weak governance, and a lack of coordination [2].

Sub-Saharan Africa has the highest burden of disease and lowest density of health workers globally [3]. In 2018, the density of doctors, nurses, and midwives per 1,000 people in the WHO African Region was 1.3, which is significantly lower than the global average of 4.6 and falls short of the minimum threshold of 4.45 recommended by the World Health Organization (WHO). Although Somalia is geographically located in the Horn of Africa, it is a member of the WHO Eastern Mediterranean Region, which makes it part of a different collaborative framework and regional healthcare strategy [4]. The needs-based shortage of health workers in Africa by 2030 is estimated at 6.1 million, the largest among all WHO regions. This shortage is partly attributed to the inadequate and inequitable production and distribution of health workers as well as the low retention and motivation of health workers in the region [5].

Somalia, a country affected by decades of civil war, political instability, and humanitarian crises, faces severe challenges in its health systems and human resources for health (HRH) [6]. The existing health system is essentially privatized and confined to major towns, leaving the poor majority in rural areas without access to affordable healthcare [7]. The national health system is fragmented, and the absence of unified health system governance has hindered national authorities’ capacity to regulate the private sector and partner with federal states to deliver services to remote areas [8]. The urban-rural population split in Somalia is approximately 47.3% urban to 52.7% rural, further challenging the equitable distribution of healthcare resources [9]. Currently, only 35% of Somalia’s population has access to essential health services [10]. The neonatal mortality rate is 36 deaths per 1,000 live births, and the under-5 mortality rate is 111 deaths per 1,000 live births [11]. The estimated maternal mortality rate is 692 per 100,000 live births [12]. Moreover, the country faces a critical shortage of skilled health professionals, with only 2.5 physicians and 4.5 nurses and midwives for every 10,000 people, falling significantly short of WHO’s minimum benchmark of 22.8 health personnel per 10,000 inhabitants [13].

Health professions education in Somalia dates back to the postcolonial period. The first nursing school was established in Hargeisa in 1966, followed by the Faculty of Medicine and Surgery in Mogadishu in 1973. These developments marked progress, but were later impacted by economic downturns and political turmoil [14]. During the Civil War, the Faculty of Medicine was looted and destroyed, and most of the medical staff and students fled the country or joined other sectors. The health system collapsed and the provision of health services was taken over by various actors, including private providers, non-governmental organizations (NGOs), and religious groups [15]. Despite these challenges, the resilience and determination of the Somali people have led to a significant resurgence in health professions education in recent years. The number of medical schools has grown from just one in 1991 to over 25 by 2024, signifying a promising development in Somalia’s healthcare system [16].

Many Somali students are highly motivated to pursue careers in the medical field because of their personal ambitions, social prestige, or religious duties. A survey conducted by the Iftiin Foundation in 2020 found that approximately 14% of graduating undergraduate students, totaling approximately 2,462 individuals, were from medical-related faculties. This number exceeds the combined number of graduates from the education and arts faculties, totaling only 1,006. The survey also revealed rising enrollment in public health colleges, reflecting an increasing demand for preventive and health-promoting strategies in Somalia [17]. However, the expansion of medical schools and student enrollment lacks effective quality control mechanisms and regulatory frameworks. The absence of regulations exposes patients to risks by enabling unqualified health professionals to practice without facing consequences, potentially leading to serious outcomes, such as misdiagnosis, incorrect treatment, infection, disability, or death [18]. The WHO has reported that Somalia has high rates of maternal and child mortality owing to poor quality healthcare and a lack of skilled birth attendants. Furthermore, the United Nations Population Fund (UNFPA) reported that over 98% of Somali women have experienced female genital mutilation, typically carried out by unqualified practitioners without proper licensing, resulting in severe health complications and risks for affected women and girls. These examples highlight the grave consequences of the lack of regulation and widespread presence of unlicensed healthcare practitioners in Somalia [19].

In 2020, the National Health Professionals Council (NHPC) Act was established to address these regulatory gaps. The NHPC aims to enhance the quality of healthcare by setting standards for health professions education and practice, ensuring that health professionals are accountable for the care they provide, and accrediting medical schools to assure quality education and practice. The mission of the NHPC is to regulate health professionals and health professional education, promote public trust in healthcare services, and improve the quality of the Somali health workforce [20]. This study aimed to provide a critical analysis of the historical factors and current situation that have contributed to the unregulated landscape of health professions education and practice in the country [12]. By examining the proliferation of medical schools, assessing the health workforce, exploring the perspectives of young physicians and healthcare students, and analyzing the regulatory framework, this study seeks to identify the implications, challenges, and opportunities for strengthening health professions education and regulation in Somalia. The findings of this study will inform policy recommendations and strategies to enhance the quality, accessibility, and equity of health care services, ultimately contributing to improved health outcomes in the Somali population.

## Methods

### Study design

This study used a mixed-methods approach, integrating both qualitative and quantitative research methodologies, to provide a comprehensive examination of the current state of health professions education and regulation in Somalia. The mixed-methods design was chosen to capitalize on the strengths of both qualitative and quantitative approaches, allowing for a more comprehensive understanding of the complex issues surrounding health professions education and regulation in the context of Somalia’s unique challenges.

### Data collection

The study on the health regulatory landscape was conducted from March 2020 to June 2021. During this period, all interviews, focus group discussions, and surveys were conducted. Between October 2023 and April 2024, we performed a literature review, mapped medical schools, consolidated the data, and developed the manuscript. Semi-structured interviews were conducted with 44 key informants, including representatives from 15 professional health schools and officials from the Ministry of Health, at both federal and state levels. The interviewees were selected based on their roles and expertise in health professions education and regulation, ensuring a representative sample across different states. Each state contributed approximately 11–16% of the total interviewees, ensuring a balanced representation.

Five focus group discussions were conducted with 25 young professionals, with each group consisting of 4 to 6 participants. These discussions explored their experiences and challenges with regard to pre-service training and employment opportunities. An online survey was administered to the students enrolled in professional health courses. The survey was distributed through university mail lists and social media platforms to maximize reach and participation. A total of 388 responses were collected, of which 35 were excluded due to non-health-related fields.

### Data analysis

Quantitative data from the surveys were entered into Excel and exported to SPSS for descriptive analysis. Qualitative data obtained from the interviews and focus group discussions were transcribed, coded, and thematically analyzed [21]. The results of both data types were triangulated to identify the main findings, gaps, and recommendations regarding the HRH regulatory landscape in Somalia. The analysis was guided by the HRH maturity model framework, which assesses the performance of health workforce regulatory functions across five stages of maturity: foundational, evolving, established, advanced, and leading [22]. Contextual factors such as the political, economic, social, and security situations in Somalia were also considered in the analysis, as they influence the HRH regulation process and outcomes. Several measures were taken to ensure the quality and rigor of this study. Triangulation was employed to cross-verify the data from multiple sources, including interviews, focus groups, and surveys. Discrepancies were resolved through discussion and consensus.

## Results

### Proliferation of medical schools

**Fig. 1:**
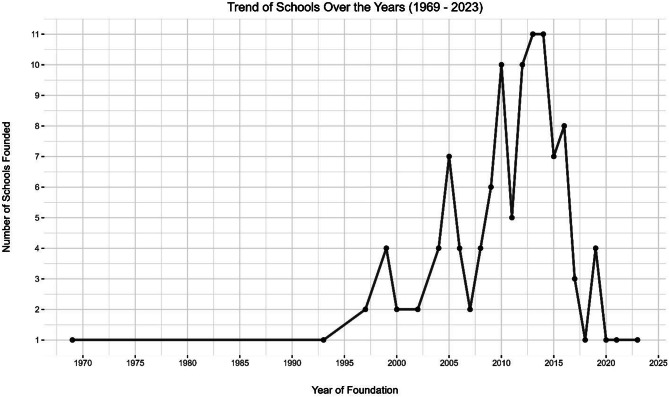
Trends in schools founded over the years (1969–2023)

**Fig. 2:**
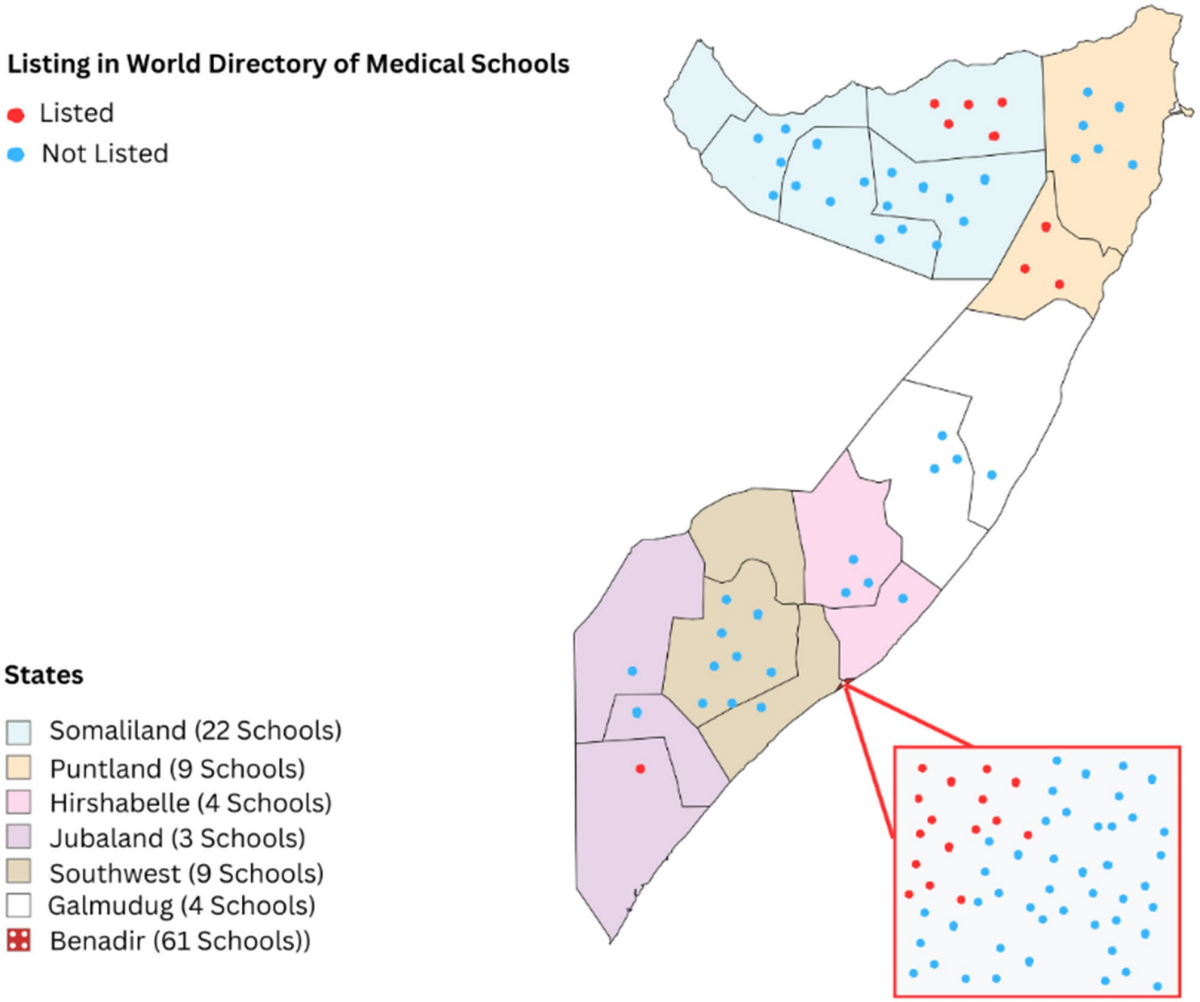
Distribution of health professions schools across different states in Somalia

This study identified 112 health professions schools across Somalia (S1) with varying distributions among different states. Approximately 54% of these institutions are in Benadir state, reflecting the concentration of educational resources in urban areas. However, schools have also been established in other states, such as Somaliland (20%), Puntland (8%), southwest (8%), Galmudug (4%), Hirshabelle (4%), and Jubaland (3%). Figure 1 shows the growth of schools in Somalia over time, with their establishment years ranging from 1969 to 2023. A significant number of these schools have been established over the last decade, highlighting a recent surge in the proliferation of medical schools. Figure 2 provides a geographical representation of the distribution of the schools in Somalia. The map is marked with red dots representing 25 schools listed in the World Directory of Medical Schools and blue dots representing those that are not listed in the directory.

### Health workforce assessment

This study evaluated the health workforce across the Benadir Regional Administration (BRA) and the states of Galmudug, Hirshabelle, Jubaland, Puntland, and Southwest. A total of 13,236 health professionals were identified, with 7,073 (53.4%) comprising of physicians, nurses, and midwives. The remaining included medical laboratory technicians, pharmacists, and public health officers. This workforce density falls below the World Health Organization (WHO) lower threshold of 2.3 skilled health professionals per 1,000 people, estimated for a population of 13 million. The distribution exhibits a pronounced urban-rural disparity, with significant shortages in both rural areas and larger urban centers at the federal and state levels. This imbalance underscores the need for targeted interventions to ensure equitable access to healthcare services across all regions in Somalia.

The physicians interviewed found their educational programs productive, especially through university partnerships with public and private hospitals in Mogadishu and month-long community medicine experiences in rural areas. However, they noted the incoherence of existing healthcare rules and regulations as well as the lack of recruitment and employment standards in these hospitals, both in the management of service providers and patients. The ratio of physicians to nurses and midwives is 1:5, compared to many African countries where the ratio is similar, and developed countries where it is closer to 1:3. This emphasizes the significant contribution of nurses and midwives to the health care system.

**Fig. 3:**
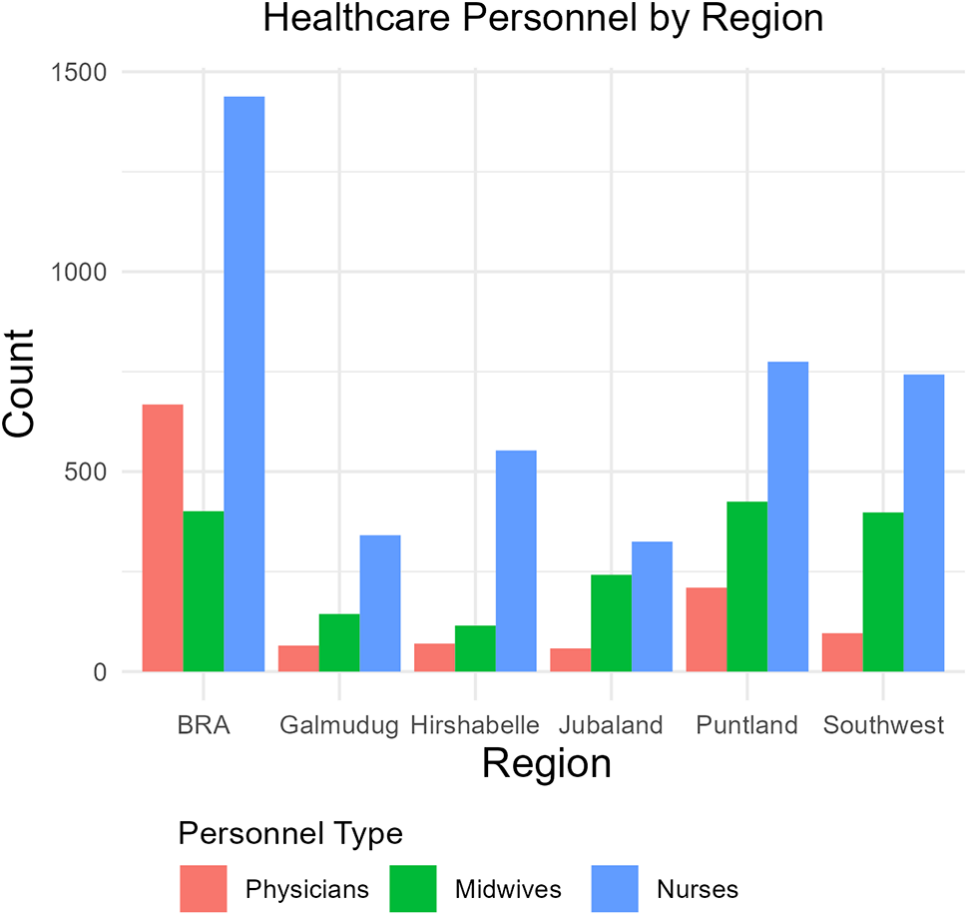
Regional Distribution of Health Professionals. (Source: Ministry of Health’s HRH report (2021)

Figure 3 highlights the disparities in the distribution of health professionals, showing the Benadir Regional Administration (BRA) with a substantial concentration of health workers: 668 physicians, 401 midwives, and 1,438 nurses. In contrast, Galmudug, Hirshabelle, and Jubaland had markedly fewer health professionals, suggesting limited healthcare service accessibility. Puntland and Southwest are moderately staffed, with Puntland having 210 physicians and 425 midwives and Southwest having 96 physicians and 398 midwives. The number of nurses was similar in both regions, with 775 in Puntland and 743 in the Southwest. Table 1 illustrates the deficits of physicians, nurses, and midwives in various levels of the health system. According to the minimum WHO staffing threshold of 23 professionals per 10,000 people, there is a requirement for 30,000 such professionals, representing a shortfall of 20,793 individuals.

In the second scenario, adopting the WHO’s preferred density rate of 44.5 professionals per 10,000 population, the need escalates to approximately 57,850 health workers, resulting in a gap of nearly 48,500. The inability of the health sector to employ all necessary graduate health workforce categories exacerbates this shortage. Despite the annual graduation of physicians, nurses, and midwives, employment opportunities remain scarce in both the public and private sectors, indicating a pressing need for policy review in health sector financing by the public sector.

**Table 1:** The WHO minimum recommended staffing threshold to support the attainment of UHC

Key HRH Categories	Currently in Service	Estimated Yearly Production	Current Need as per WHO Minimum Density Rate of 23 per 10,000 Population	Current Need as per WHO Revised Density Rate of 44.5 per 10,000 Population	HRH Gap as per WHO Indicated two Coverage Scenarios	Time Projected for Bridging the Current two Coverage Scenarios Gap in Years*
Physicians	1167	800	Current density: 5.4 per 10,000 population. Estimated need of the three categories as per 23 per 10,000 population is: 29,900. Attainable in 8years with the higher production rate	Current density: 5.4 per 10,000 population Estimated need of the three categories to achieve 44.5 per 10,000 population is: 57,850. Attainable in 25 years with the higher production rate	22,827& 50,777	8 and 17 years respectively.
Registered Nurses	4175	1400				
Registered Midwives	1731	780				
**Totals**	**7**,**073**	**3**,**000**	**29**,**900**	**57**,**850**		

Source: Ministry of Health’s HRH report (2021)

*The projected gap was measured against a population of approximately 13 million. These estimates did not factor in the additional health workforce need resulting from the 2.9% annual population growth rate in Somalia, reflecting an additional 12,000 to 24,000 of these three professional categories, depending on which of the two thresholds to consider

### Focus group discussion with young physicians

The physicians interviewed expressed dissatisfaction with employment opportunities after graduation, highlighting the need for strong connections and family linkages to secure jobs. One participant stated, ‘Without connections, it is almost impossible to find a decent job in the healthcare sector.’ This sentiment was echoed by others, who noted a lack of fair recruitment practices. Another interviewee remarked, ‘Most of my colleagues who got jobs had some form of connection within hospital administration.

Physicians indicated that the private sector is equally affected by the need for strong connections and family linkages for job offers. On many occasions, the only opportunity offered is voluntary practice without financial compensation, which many accept as advancing their professional skills. This makes the private sector less attractive. One participant jointly established a small clinic with a fellow physician, and said that they were managed reasonably. However, his colleagues indicated that this avenue of employment is only possible for a limited number of people who can afford the upfront tangible investment for establishing a clinic.

The physicians interviewed felt that the above reasons severely limited their professional opportunities, in many cases compelling graduates to change their profession and engage in non-health areas or opt for a life as a housewife, as is the case for many female health professionals.

### Employment and education perception survey

In less than two weeks, 388 students from across the country responded, demonstrating their interest in regulations. After excluding 35 responses from non-health-related fields, we analyzed data from 353 students, most of whom were enrolled in priority courses, such as nursing (33%), Public Health (22%), Medicine and Surgery (15%), midwifery (14%), and medical laboratories (10%). Approximately 80% of respondents were satisfied with the quality of their training. However, 56% of the students from the BRA who responded were dissatisfied. About 70% of students would prefer to study in Somalia, thus building the case for standardization of academic training experiences across the country.

In contrast with the practicing physicians interviewed, almost 90% of the students perceived their employment opportunities to be good, and 80% agreed to consider working in rural areas if given a chance. Close to 30% of BRA and 15% of Jubaland students do not consider their employment opportunities to be good. However, all agreed that the existing workforce demanded more opportunities for practical training, professional development, employment, and stricter regulations. Several challenges in working in rural areas, dominated by a lack of security and terrorism, were highlighted.

### Insufficient HRH team capacity for regulatory functions

Interviewees described that the HRH team’s capacity at both federal and state levels was insufficient to carry out all the regulatory and non-regulatory activities required. At both the federal and state levels, there are not enough workers in teams. In addition, many HRH team members do not receive the necessary training to perform their work optimally. Most teams have little budget beyond their salaries, meaning that there is little or no budget for communication, printing, travel, IT, or training.

### Regulatory framework

The interviewees stated that the insufficiency of a functional health regulatory framework was a challenge in establishing medical regulations. They described the institution of a health regulatory framework as an important milestone in standardizing professional health education in both pre-service and in-service training and practice. Interviewees described that, in the absence of a formal regulatory framework during the past decades, loose self-regulation has been the norm. Several professional groups, including medical and dental associations, nursing and midwifery associations, pharmacists, and other professional groups, have organized their members to improve access to and quality of healthcare services, set standards of best practice, promote health, prevent diseases, and deliver curative and rehabilitative services following the established health sector service delivery guidelines.

These associations were also formed to protect and guard their respective professional interests in terms of regulation, licensing, Continuing Medical Education (CME), continuing professional development (CPD), preserving ethical norms, setting practice standards, and representing their professions at the national and international levels. In the absence of a binding national or state-level regulatory framework, professional associations sustained a voluntary registration system with weak licensing authorities, while having little or no influence on the pre-service regulation of their respective educational institutions.

### Lack of licensing exams

Interviewees stated that credentials obtained from educational institutions act as proxy licensing permits, enabling new graduates to seek employment opportunities in the healthcare system. Neither the educational institutions nor the health facilities that would potentially employ these newly graduated professionals are registered or licensed, although most educational institutions are voluntarily registered by the Ministries of Higher Education and/or by competent state-level government authorities where they operate formally.

### Lack of continuing professional development

This study found a significant gap in the professional development of health workers in Somalia, with no existing mandates for Continuing Professional Development (CPD). This lack of CPD requirements is further compounded by the general scarcity of training opportunities, which hinder the ongoing enhancement of skills and knowledge among health professionals. However, the introduction of the National Health Professionals’ Council (NHPC) Act marks a pivotal change, acknowledging the importance of CPD for the entire health workforce. This recognition by the NHPC paves the way for implementing structured CPD programs that are essential for maintaining high standards of care and ensuring that proficient and up-to-date professionals deliver health services.

### Lack of accreditation of Pre-service Education (PSE)

School representatives and federal and state representatives generally agreed that the federal government, in close collaboration with the states, should accredit pre-service education for the five main cadres (physicians, nurses, midwives, pharmacists, and dentists), and that the states should accredit pre-service education for all other cadres. There was agreement on the need to harmonize all other cadres across the states to allow health worker movement and avoid confusion. The schools indicated that they were mainly self-regulating and tended to have little contact with either the Ministry of Health or Ministry of Education. The schools mentioned that regulatory uncertainty made it difficult to establish new schools and expand the existing programs. It was also stated that because there were no set standards for the length of training or competencies for each cadre, the competencies of graduates of a given profession differed greatly from school to school, creating uncertainty in the labor market.

### Professional misconduct and disciplinary powers

Interviews with key informants revealed a prevalent issue of misconduct involving individuals known as charlatans. Charlatans falsely present themselves as licensed health workers, particularly physicians and pharmacists, despite the lack of official credentials. Key informants generally recognized dual practices, wherein health workers employed in public institutions also operate private practices, as widespread and not considered problematic. In the Somali health system, dual practices help health workers supplement their low public salaries and maintain public employment.

## Discussion

The findings highlight Somalia’s health professions education and regulatory framework, which is crucial for achieving Sustainable Development Goals and universal health coverage. Despite a notable increase in the number of health professions schools, regulatory mechanisms have struggled to keep pace, resulting in concerns about quality and standardization. The establishment of the National Health Professionals’ Council (NHPC) Act in 2020 marks a significant step toward addressing these challenges. The NHPC began operating on September 21, 2023, and aims to enhance the quality of healthcare by setting standards for health professions education and practice, ensuring that health professionals are accountable for the care they provide, and accrediting health professions schools to assure quality education and practice. The mission of the NHPC aligns closely with the findings of this study. For instance, the NHPC aims to set standards for health professions education and practice. This directly addresses the study’s finding of significant disparities in the distribution of healthcare professionals, with shortages in both rural areas and larger urban centers. By establishing and enforcing these standards, the NHPC can help mitigate these imbalances and ensure that health professionals are adequately prepared to serve all regions of Somalia. This mission contrasts with the current situation where the absence of strong regulatory mechanisms has led to inconsistent quality in health professions education.

Accreditation of institutions by the NHPC is another critical objective that aligns with the findings of this study. The study found that only 25 of the 112 schools were listed in the World Directory of Medical Schools (WDOMS). However, it is important to note that being listed in the WDOMS does not equate with accreditation. This listing merely confirms the existence of the medical school but does not denote recognition or accreditation. The NHPC’s role in accrediting these schools will help ensure that they meet national and international standards, thereby improving the quality of education and healthcare services. This effort is essential to address the study’s concern about the lack of quality control mechanisms in the expansion of health professions schools. Furthermore, the NHPC’s responsibility to regulate health professional practices to ensure accountability and quality of care directly responds to the issues highlighted in the study. The study noted the incoherence of existing healthcare rules and regulations and the lack of recruitment and employment standards. By establishing clear regulations and standards for practice, the NHPC can improve the consistency and quality of healthcare services across the country. This regulatory role is critical in addressing the professional misconduct and unlicensed practices that the study identified as widespread problems undermining public confidence in the healthcare system.

The NHPC also emphasizes the importance of continuing professional development for health professionals, which aligns with the findings of this study. This study found that many physicians appreciated the productivity of their educational programs, particularly through partnerships with hospitals and community medicine experiences. The NHPC can build on these strengths by promoting continuous learning and professional growth, ensuring that health professionals remain updated with their latest knowledge and skills. Enhancing public trust in healthcare services is another objective of the NHPC, which aligns with the findings of this study. This study highlighted the grave consequences of the lack of regulation and the presence of unlicensed healthcare practitioners. By setting and enforcing high standards for health professions education and practice, the NHPC can help build public trust in the healthcare system. This is critical for improving health outcomes and ensuring that the Somali population has confidence in the healthcare services that they receive.

Assessment of the health workforce reveals a significant shortage of skilled health professionals, with the current density falling below the World Health Organization’s minimum threshold. The pronounced urban-rural disparity in the distribution of health workers further exacerbates the challenges in healthcare service delivery. The ratio of physicians to nurses and midwives (1:5) emphasizes that nurses and midwives play a crucial role in the healthcare system. However, the overall shortage of healthcare providers, as evidenced by the gap analysis based on the WHO’s recommended density rates, calls for urgent policy interventions to address this critical issue.

These findings are consistent with those of studies conducted in other countries. A study in Kenya reported a similar shortage of healthcare workers, with a density of 1.5 physicians and 7.9 nurses and midwives per 10,000 population, falling short of the WHO’s recommended threshold [23]. Similarly, a study in Tanzania highlighted the uneven distribution of healthcare workers, with a higher concentration in urban areas and a scarcity in rural regions [24]. These studies underscore the common challenges faced by the healthcare systems in East Africa, emphasizing the need for collaborative efforts to address the shortage and maldistribution of healthcare professionals. Focus group discussions with young physicians have shed light on employment challenges faced by medical graduates. The lack of fair and transparent recruitment processes coupled with the need for strong connections and family linkages hinders the entry of qualified professionals into the workforce. Limited employment opportunities in both the public and private sectors, along with financial constraints in establishing independent clinics, force many graduates to seek alternative careers or to remain unemployed. This brain drain and underutilization of medical expertise poses a significant threat to the healthcare system’s sustainability and effectiveness.

Similar challenges have also been reported in other countries. A study in Uganda found that medical graduates faced difficulties securing employment because of limited job opportunities and nepotism during the recruitment process [25]. The study also highlighted the financial barriers faced by young physicians in establishing private practices, leading to a significant proportion of graduates opting for nonmedical careers. These findings emphasize the need for policy interventions to create an enabling environment for the absorption of medical graduates into the health care workforce. The Employment and Education Perception Survey provided valuable insights into the perspectives of healthcare students. While most respondents expressed satisfaction with the quality of their training, dissatisfaction among students in the Benadir Regional Administration (BRA) warrants further investigation. The strong preference for studying within Somalia highlights the need for the standardization of academic training experiences across the country. Perceived employment opportunities among students contradict the experiences of practicing physicians, indicating a potential mismatch between expectations and reality. The willingness of students to work in rural areas, given the right conditions, presents an opportunity to address the urban-rural disparity in healthcare service provision.

Analysis of the Somali HRH regulation landscape revealed several critical challenges. The insufficient capacity of HRH teams at both federal and state levels hinders the effective implementation of regulatory functions. The absence of a comprehensive regulatory framework has led to reliance on self-regulation by professional associations, which lack the necessary authority and influence to enforce standards and ensure quality. The lack of licensing exams and continuing professional development opportunities further compromises the competence and skills of healthcare professionals. However, these regulatory challenges are not unique to the Somalia. A study in Rwanda identified similar gaps in the regulatory framework, including the absence of a comprehensive licensing system and a limited capacity for regulatory oversight [26]. The study emphasized the importance of strengthening regulatory bodies and developing clear guidelines for professional practice. These findings highlight the need for a robust regulatory framework to ensure the quality and safety of health care services. The absence of accreditation for pre-service educational institutions creates inconsistencies in the quality and duration of training programmes, leading to variations in graduate competencies. A study in Ethiopia reported similar challenges, with a lack of standardization in health professions education curricula and limited quality assurance mechanisms. This study recommends the establishment of a national accreditation system to ensure the quality and relevance of health professions education programs [27]. These findings underscore the importance of accreditation for ensuring the production of competent healthcare professionals.

The prevalence of professional misconduct, particularly that involving charlatans, undermines public trust in the healthcare system and poses risks to patient safety. The widespread dual practice, which supplements low public salaries, requires careful monitoring and regulation to ensure the quality of care. Similar challenges have also been reported in other studies. A study in Kenya found that a lack of regulation and oversight of private healthcare facilities contributed to the proliferation of unqualified practitioners and compromised patient safety [28]. The study emphasized the need for strengthened regulatory mechanisms to curb professional misconduct and ensure the delivery of quality healthcare services. This study highlights significant challenges in the Somali health professions education system, including the lack of international accreditation and national standardization. Only 25 medical schools were listed in the World Directory of Medical Schools, indicating a lack of international recognition. This lack of standardization has resulted in varying curricula, inconsistent training quality, and differences in graduate competencies across medical schools in Somalia. Addressing these issues requires establishing a robust national accreditation system to ensure uniform educational standards and to improve the overall quality of medical training. Investment in capacity building of HRH teams at both federal and state levels is crucial to enable effective regulatory oversight. The implementation of continuing professional development programs, as mandated by the NHPC Act, will help maintain and enhance the skills and knowledge of healthcare professionals. Collaboration between the Ministry of Health, Ministry of Education, and professional associations is essential to harmonize training standards and ensure the alignment of educational programs with the healthcare system’s needs.

Efforts to address these challenges include enactment of the National Health Professionals’ Council (NHPC) Act, which aims to support regulatory oversight and standardize health professional education and practice. However, capacity constraints, inadequate professional development opportunities, and absence of licensing examinations continue to hinder progress in this sector. There is a significant lack of stakeholder engagement, which has resulted in outdated curricula failing to align with the country’s healthcare needs and priorities. The lack of a structured approach involving key stakeholders, such as healthcare providers, policymakers, educators, and community representatives, has led to insufficient resources and a disconnection between educational content and the realities of healthcare delivery in Somalia.

Our findings are consistent with those of previous studies, which have highlighted the importance of stakeholder engagement in curriculum development. One study conducted in Namibia emphasized that curriculum development is an ongoing process involving a wide range of stakeholders, including professional educators, consultants, administrators, teachers, students, parents, politicians, and community members [29]. In contrast, a study conducted in Kenya found that Kenya successfully implemented stakeholder engagement models in its health professions education system. A significant majority of institutional heads and faculty members in Kenya reported having written guidelines for curriculum review, ensuring that educational content remains responsive to national health needs and aligns with regulatory standard [30]. This finding suggests that by adopting a stakeholder-driven approach like Kenya’s, Somalia could rejuvenate its health professions education system and ensure that curricula are consistently updated, relevant, and adequately prepare students to effectively tackle healthcare challenges.

This study has several limitations, including the potential for selection bias in the recruitment of key informants and reliance on self-reported data in surveys and interviews, which may be subject to social desirability bias. Additionally, the generalizability of the findings may be limited because of the specific context of Somalia. Another limitation is the lack of contact with representatives from the Ministry of Education, Culture and Higher Education, which could have provided a more comprehensive understanding of the regulatory and educational landscape. Future research could address these limitations by employing random sampling techniques, triangulating self-reported data with objective measures whenever possible, and including insights from the Ministry of Education to enrich the study’s findings.

## Conclusion

This study provides an analysis of the current state of health professions education and its regulations in Somalia. The findings highlight the significant challenges faced by the healthcare system, including the proliferation of health professions schools without adequate quality control, a critical shortage and maldistribution of skilled health professionals, the absence of a comprehensive regulatory framework, and the prevalence of professional misconduct. The rapid growth of health professions schools in Somalia reflects the growing demand for health professions education. However, the lack of international accreditation for most of these schools raises concerns regarding the quality and standardization of medical training. The concentration of health professions schools in urban areas, particularly in Benadir, underscores the need for a more equitable distribution of educational resources to ensure access to high-quality health professions education nationwide. Assessment of the health workforce reveals a significant shortage of skilled health professionals, with the current density falling below the World Health Organization’s minimum threshold. The pronounced urban-rural disparity in the distribution of health workers further exacerbates the challenges in healthcare service delivery. The establishment of the National Health Professionals’ Council (NHPC) Act in 2020 marks a significant step towards a comprehensive regulatory framework. The NHPC should prioritize the accreditation of health professions schools, ensuring that all institutions meet the required standards for quality education. Investment in capacity building of HRH teams at both federal and state levels is crucial to enable effective regulatory oversight. Collaboration between the Ministry of Health, Ministry of Education, and professional associations is essential to harmonize training standards and ensure the alignment of educational programs with the healthcare system’s needs. Strategies to address the urban-rural disparity in healthcare service provision should be developed, including incentives for healthcare professionals to work in underserved areas. The government should also explore innovative financing mechanisms to support the establishment of healthcare facilities and employment of healthcare professionals in rural regions. Furthermore, public awareness campaigns should be launched to educate the population about the importance of seeking care from licensed healthcare professionals and combat the prevalence of charlatans. The regulation of dual practices should be strengthened to ensure that healthcare professionals maintain high standards of care in both public and private settings. By addressing the challenges identified in this study and adopting a comprehensive approach to healthcare workforce development and regulation, Somalia can make significant strides towards achieving universal health coverage and improving the health outcomes of its population.

## Electronic supplementary material

Below is the link to the electronic supplementary material.

Supplementary Material 1

## Data Availability

The datasets used in the current study are available from the corresponding author upon reasonable request.
